# Prevalence of Viral Hepatitis Antibodies Among Alcoholics in Croatia: A Single Center’s Results

**DOI:** 10.3390/antib15020020

**Published:** 2026-02-25

**Authors:** Maja Vilibić, Klara Barbić, Maja Bogdanić, Snježana Židovec-Lepej, Ana Matošić, Ana Sanković, Dalibor Karlović, Leona Radmanić Matotek, Nataša Kutela, Sergej Nadalin, Ema Borko, Vladimir Savić, Ljubo Barbić, Marija Santini, Hrvojka Janković, Vladimir Stevanović, Tatjana Vilibić-Čavlek

**Affiliations:** 1 Department of Psychiatry, “Sestre Milosrdnice” University Hospital Center, 10000 Zagreb, Croatia; ana.matosic@kbcsm.hr (A.M.); dalibor.karlovic@kbcsm.hr (D.K.); 2 School of Medicine, Catholic University of Croatia, 10000 Zagreb, Croatia; sergej.nadalin@medri.uniri.hr; 3 Statistics Concentrator, Harvard University, Cambridge, MA 02139, USA; klarabarbic@college.harvard.edu; 4 Department of Virology, Croatian Institute of Public Health, 10000 Zagreb, Croatia; maja.bogdanic@hzjz.hr (M.B.); emaborko01@gmail.com (E.B.); hrvojka.jankovic@hzjz.hr (H.J.); 5 School of Medicine, University of Zagreb, 10000 Zagreb, Croatia; 6 Department of Immunological and Molecular Diagnostics, University Hospital for Infectious Diseases “Dr. Fran Mihaljević”, 10000 Zagreb, Croatia; szidovec@gmail.com (S.Ž.-L.); leona.radmanic@gmail.com (L.R.M.); nkutela@bfm.hr (N.K.); 7 Department of Psychiatry and Medical Psychology, School of Dental Medicine, University of Zagreb, 10000 Zagreb, Croatia; 8 Department of Microbiology, University of Applied Health Sciences, 10000 Zagreb, Croatia; asankovic@zvu.hr; 9 Department of Psychiatry, General Hospital “Dr. Josip Benčević”, 35000 Slavonski Brod, Croatia; 10 Poultry Center, Croatian Veterinary Institute, 10000 Zagreb, Croatia; v_savic@veinst.hr; 11 Department of Microbiology and Infectious Diseases with Clinic, Faculty of Veterinary Medicine, University of Zagreb, 10000 Zagreb, Croatia; ljubo.barbic@vef.hr (L.B.); vladostevanovic@gmail.com (V.S.); 12 Department for Infections in Immunocompromised Patients, University Hospital for Infectious Diseases “Dr. Fran Mihaljević”, 10000 Zagreb, Croatia; msantini@bfm.hr

**Keywords:** alcoholics, hepatitis A, hepatitis B, hepatitis C, hepatitis E, prevalence, risk factors, Croatia

## Abstract

Background/Objectives: Viral hepatitis A–E represents a significant public health problem. Data on the prevalence of viral hepatitis markers among alcoholics are inconsistent. Methods: The study included 151 patients treated for alcohol abuse in one Croatian center. The control group consisted of 110 individuals from the general population tested for a routine check-up. The prevalence of viral hepatitis markers was assessed using serology and molecular methods. Results: The prevalence rates of hepatitis markers among patients were as follows: anti-HAV, 15.2%; anti-HBs, 11.9%; anti-HBc/anti-HBs, 2.6%; anti-HCV, 4.0%; and anti-HEV, 14.6%. HCV RNA was detected in one patient (0.6%). Compared with the control group, patients showed significantly higher HCV seroprevalence (4.0 vs. 0%), while the prevalence of other hepatitis markers did not differ significantly between the groups. The anti-HAV prevalence was associated with age (from 0% in patients aged <40 years to 42.9% in patients aged 60+ years), employment status (highest among retired individuals at 46.2%), and age of occasional alcohol consumption (highest seroprevalence of 26.3% in those who reported consumption between 22 and 25 years). The association between anti-HEV and educational level was of borderline significance. Logistic regression showed that older and retired patients and those who consumed alcohol occasionally between 22 and 25 years showed higher odds for HAV seropositivity (OR = 11.454–49.400, OR = 6.857, and OR = 4.464, respectively). Patients with university degrees were at lower risk for HEV seroprevalence (OR = 0.083). Conclusions: Alcoholic patients showed a higher HCV seroprevalence than the general population, while the prevalence of other viral hepatitis markers did not differ between the groups. Further studies on a larger cohort of patients are needed to confirm these findings.

## 1. Introduction

Viral hepatitis represents a major global health challenge and remains a significant cause of morbidity and mortality worldwide. The primary hepatotropic viruses, hepatitis A (HAV), hepatitis B (HBV), hepatitis C (HCV), and hepatitis E (HEV), can cause a wide spectrum of clinical manifestations, ranging from asymptomatic infection to acute, chronic, or fulminant disease [1].

HAV, a member of the *Picornaviridae* family, *Hepatovirus* genus, is a global pathogen responsible for an estimated 1.4 to 1.5 million hepatitis cases each year [2]. The virus is primarily transmitted via the fecal–oral route. The transmission is associated with inadequate sanitation and poor hygiene, resulting in significant variations in HAV infection prevalence across populations and geographic regions [3], ranging from 10% in developed Scandinavian countries to 100% in Asian and African countries [4].

HBV belongs to the family *Hepadnaviridae*, genus *Orthohepadnavirus*. According to the 2024 World Health Organization (WHO) report, about 254 million people worldwide are estimated to be living with chronic HBV infection [5]. In high-endemic regions, perinatal and early childhood transmission are common. In contrast, in the low-endemic areas, transmission is more often through adult high-risk behaviors (sexual contact, exposure to contaminated needles). Sub-Saharan Africa, particularly the Western and Central regions, is highly endemic for HBV, in contrast to the substantially lower burden observed in the Americas and Western Europe. Across Europe, HBsAg prevalence is generally low in Western and Northern countries (<0.5–1%), whereas several Eastern and South-Eastern countries report higher levels, ranging from 1 to 2% [6].

HCV, a member of the *Flaviviridae* family, *Hepacivirus* genus, infects about 2–3% of the global population. According to recent estimates, about 50 million people worldwide are living with chronic HCV infection that can result in severe liver damage, such as cirrhosis and hepatocellular carcinoma, contributing to an estimated 350,000–400,000 deaths annually [7]. The virus spreads through blood transfusions, hemodialysis, improperly sterilized medical equipment, organ transplantation, intravenous drug use (IDU), and, less frequently, sexual contact [8]. Within the European Union/European Economic Area (EU/EEA), the estimated prevalence in 2019 was 0.5%, although it varied considerably, with higher rates observed in the Eastern EU/EEA (0.88%) than in Western and Northern Europe (0.27–0.41%) [9].

HEV belongs to the genus *Orthohepevirus*, *Hepeviridae* family. An estimated 20 million HEV infections occur annually worldwide, causing 3.3 million symptomatic cases and ~44,000 deaths. Over 60% of documented cases occur in South/East Asia, China, and North/Sub-Saharan Africa, where large waterborne outbreaks are common. In these regions, contaminated drinking water is a common source of outbreaks, with HEV genotypes 1 and 2 being responsible for the majority of infections [10]. In developed regions, HEV infections are caused by zoonotic HEV-3 genotype, and the transmission mainly occurs through consumption of undercooked pork, wild boar, or deer meat [11].

Some studies indicate that individuals with alcohol use disorders exhibit a higher prevalence of viral hepatitis compared with the general population [12,13]. In Croatia, alcohol consumption is widely regarded as a socially accepted behavior characterized by a high level of societal tolerance [14]. According to Eurostat data (European Health Interview Survey, 2019), 10.2% of the Croatian population aged 15 and older reported drinking alcohol daily, compared to 17.9% consumed alcohol weekly, and 21.0% consumed alcohol monthly [15]. Several epidemiological studies conducted in Croatia analyzed the seroprevalence of viral hepatitis markers in different exposed and non-exposed populations [8,16,17,18,19,20,21]. The seroprevalence differed significantly between the groups and geographic regions. However, data on the prevalence of viral hepatitis in alcoholics are scarce. Given that alcoholics represent a potential risk group due to alcohol-related liver damage, this study aimed to analyze the prevalence of HAV, HBV, HCV, and HEV markers in patients treated in one clinical hospital center in Croatia. Furthermore, predictors for HAV and HEV seropositivity were analyzed.

## 2. Materials and Methods

### 2.1. Patients

The study included 151 patients with alcohol abuse treated at the “Sestre Milosrdnice” University Hospital Center in Zagreb from June 2024 to December 2025. In the patient group, there were 128 (84.8%) males and 23 (15.2%) females aged 25–80 years. The control group consisted of 110 participants (65 males, 59.1%, and 45 females, 40.9%) aged 18–77 years from the general population, tested as part of a routine check-up (preoperative screening, medically assisted reproduction) at the Croatian Institute of Public Health. Controls were matched to the patient group by age to minimize age-related confounding. No significant difference (*p* = 0.246) was found between the age of patients (median 46 years, IQR = 41–54) and controls (median 45 years, IQR = 35–56). None of the patients or controls reported a history of blood transfusions, IDU, or homelessness. Some patients were temporarily detained in police facilities as a security measure; however, none reported a history of imprisonment. Eighteen patients were vaccinated against hepatitis B, while none were vaccinated against hepatitis A.

For patients, data on sociodemographic characteristics (sex, age, educational level, employment), alcohol-related risk behaviors, and other potential predictors, such as history of traveling/long stays abroad and eating habits (HEV), were collected using a questionnaire (Table 1 and Table 2). For the control group, no data were available, except for demographic characteristics.

Potential predictors for HEV infection included professional exposure (hunter; 1.3% of patients), consumption of game meat (60.3%) and pork liver (70.9%), as well as pig farming (2.0%). Traveling or long-term stay abroad was reported by 17.2% of participants (Table 2).

### 2.2. Laboratory Testing

Detection of viral hepatitis antibodies and antigens was performed using commercial enzyme-linked fluorescent assays (ELFA; Vidas Kube, Marcy l′Etoile, France) (Table 3).

Anti-HCV-positive samples were further tested for HCV RNA. HCV RNA levels in serum were quantified using the Xpert HCV Viral Load real-time PCR test (Cepheid, Maurens-Scopont, France), with a linear range of 1.0 to 8.0 log_10_ IU/mL. Samples that tested positive for HEV antibodies were confirmed using a commercial immunoblot assay using highly purified recombinant HEV antigens: O2N (N-terminal part of the ORF 2 protein) genotype 1/3, O2C (C-terminal part of ORF 2 protein) genotype 1/3, O2M (middle part of the ORF 2 protein) genotype 1, O3 (ORF 3 protein) genotype 1/3 (HEV recomLine, Mikrogen Diagnostik, Neuried, Germany).

### 2.3. Statistical Analysis

Descriptive statistics were used to summarize sociodemographic characteristics and alcohol-related risk behaviors in the patient survey dataset (*n* = 151). Missing data were excluded from the analysis. Overall and stratified seroprevalence were estimated with exact 95% confidence intervals (CI; Clopper–Pearson) using binomial methods. For comparisons with reference prevalence, exact binomial tests were performed using the control-group seroprevalence as the null proportion (p_0_ = 0.12). Associations between each predictor and seroprevalence (anti-HAV and anti-HEV) were assessed using univariable binomial logistic regression models. When standard logistic regression exhibited (quasi)complete separation leading to unstable estimates, Firth penalized logistic regression was applied (using the logistf package), with penalized-likelihood odds ratios (ORs), 95% CI, and *p*-values reported. For multi-level predictors, one category served as the reference, and ORs with 95% Wald CI were reported for pairwise contrasts. Age and alcohol quantity were evaluated using prespecified categories. Observed seroprevalence by predictor level was visualized as bar plots with exact 95% CI. For the predictors identified as significant in global testing (employment and occasional alcohol consumption), a heat map was used to display anti-HAV seroprevalence across joint strata of employment and occasional alcohol consumption. Figures were produced using ggplot2. All tests were two-sided with α = 0.05, and *p* < 0.05 was considered statistically significant. Since the sample consisted of only 151 patients, no asymptotic tools were used for statistical analysis. Exact tests were used to account for the small sample size. Analyses were conducted in R version 4.4.2 (R Foundation for Statistical Computing, Vienna, Austria).

## 3. Results

The prevalence of viral hepatitis markers in patients and controls is presented in Table 4. In the patient group, the seroprevalence of anti-HAV was 15.2%, and that of anti-HEV was 14.6%. Anti-HBc/anti-HBs was detected in 2.6% of patients and anti-HCV in 4.0%, while HCV RNA was detected in one patient (0.6%). The prevalence of anti-HCV was significantly higher in patients than in controls (4.0 vs. 0%, *p* = 0.042). No significant difference was observed in the anti-HAV, HBsAg, anti-HBc/anti-HBs, and anti-HEV between patients and controls. Post-vaccinal HBV immunity (anti-HBs positive) was detected in 11.9% of patients and 20.0% of controls (*p* = 0.073).

**Figure 1:**
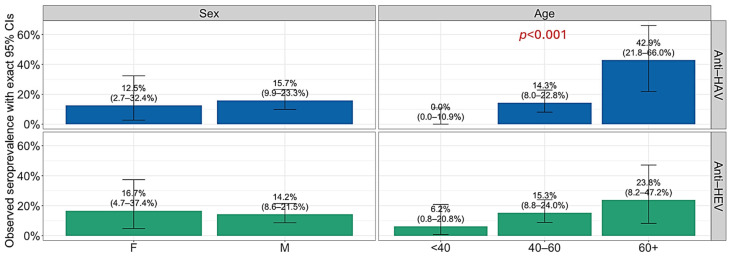
Prevalence of anti-HAV and anti-HEV antibodies in alcoholics by sex and age (years). Boxes represent seroprevalence rates with 95% confidence intervals (CIs).

**Figure 2:**
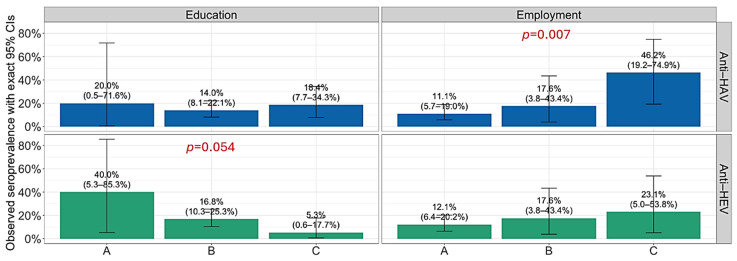
Prevalence of anti-HAV and anti-HEV antibodies in alcoholics by educational level and employment status. Boxes represent seroprevalence rates with 95% confidence intervals (CIs). Education: A = primary school, B = high school, C = university; Employment: A = employed, B = non-employed, C = retired.

**Figure 3:**
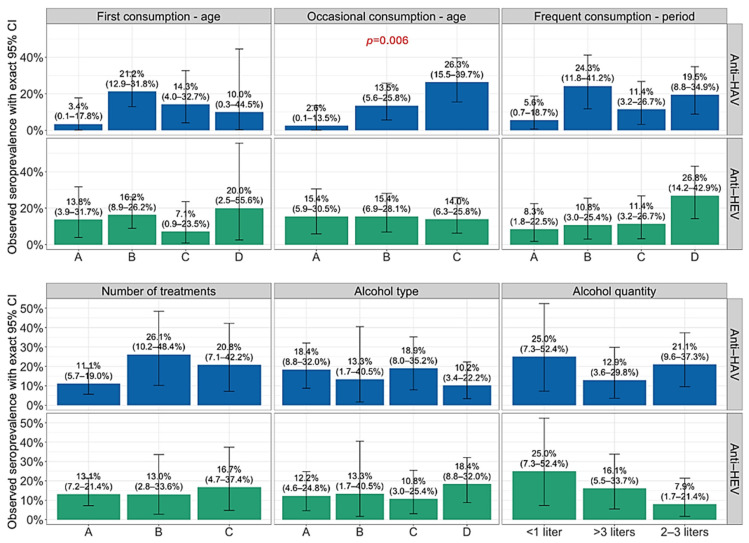
Prevalence of anti-HAV and anti-HEV antibodies in alcoholics by alcohol-related risk behavior. Boxes represent seroprevalence rates with 95% confidence intervals (CIs). First alcohol consumption (age): A ≤ 14 years, B = 14–18 years, C ≥ 18–22 years, D ≥ 22 years; occasional alcohol consumption (age): A = 14–18 years, B ≥ 18–22 years, C ≥ 22–25 years; frequent alcohol consumption (period): A ≤ 5 years, B = 5–10 years, C ≥ 10–15 years, D ≥ 15 years; number of treatments: A = first, B = second; C = third or more; alcohol type: A = beer, B = wine, C = whiskey, D = combined.

HAV and HEV seropositivity by sociodemographic characteristics and alcohol-related risk predictors are presented in Figure 1, Figure 2 and Figure 3. Analyzing the HAV seroprevalence by sociodemographic characteristics of patients, significant differences were observed according to age, employment status, and age of occasional alcohol consumption. Retired patients showed significantly higher seropositivity (46.2%) compared to employed (11.1%) and non-employed patients (17.6%), respectively (*p* = 0.007). Patients who reported occasional alcohol consumption between >22 and 25 years were more often seropositive (26.3%) than those who consumed alcohol at a younger age (2.6 and 13.5%; *p* = 0.006) (Figure 3). The association of HEV seropositivity with educational level was of borderline significance (*p* = 0.054), with a decline in the seroprevalence from 40.0% in patients with primary education to 5.3% in patients with university graduation (Figure 2). A progressive increase in seropositivity with age was observed. These differences were statistically significant for HAV (*p* < 0.001), but not for HEV (*p* = 0.189) (Figure 1). Other analyzed parameters were not associated with anti-HAV and anti-HEV seropositivity.

No significant differences in seroprevalence were observed in patients who reported traveling (HAV 11.5 vs. 16.0%; *p* = 0.685; HEV 11.5 vs. 15.2%, *p* = 0.728) or consumption of game meat/pork liver (HAV 13.2 vs. 18.3%, *p* = 0.677; HEV 14.3 vs. 15.0%, *p* = 0.189).

Results of global association tests and global likelihood ratio tests for anti-HAV and anti-HEV seropositivity are presented in Table 5, and the results of the univariable logistic regression for statistically significant variables are presented in Table 6.

Age, employment status, and age at the time of occasional alcohol consumption were associated with HAV seropositivity (Table 5). Patients aged 40–60 and 60+ years had higher odds than patients <40 years (OR = 11.154, *p* = 0.015 and OR = 49.400, *p* < 0.001, respectively). Retired patients had higher odds of being anti-HAV seropositive (OR = 6.857, *p* = 0.002) (Table 6). In addition, patients who consumed alcohol frequently for 5–10 years had higher odds compared to those who consumed alcohol for less than 5 years (OR = 5.464, *p* = 0.038).

In the analysis of predictors of HEV seropositivity, educational level was identified as a significant predictor of anti-HEV seroprevalence. Patients with a university-level education were less likely to be anti-HEV seropositive compared with those with primary school education (OR = 0.083, *p* = 0.033) (Table 6).

**Figure 4:**
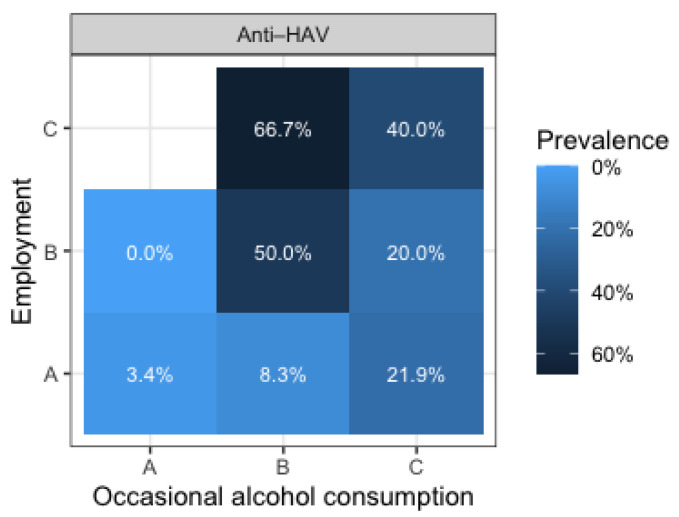
Predicted anti-HAV prevalence according to significant predictors (Employment: A = employed, B = non-employed, C = retired; occasional alcohol consumption (age): A = 14–18 years, B ≥ 18–22 years, C ≥ 22–25 years; age: <40 is empty).

The predicted HAV seroprevalence according to significant predictors is presented in Figure 4. The heat map shows the predicted HAV seroprevalence according to employment and occasional alcohol consumption (age), based on the results of logistic regression. Although age was also a significant predictor, it is not shown because of the small number of tested patients, due to which some cells are empty, and many other cells were 0% or 100%, with all of that making the graph uninstructive (cell refers to a combination of levels of three significant predictors). Predicted HAV seroprevalence was higher in individuals who started occasionally consuming alcohol at age >18–22 years (50.0% non-employed, 66.7% retired) than in those who started at age >22–25 years (20.0% non-employed, 40.0% retired).

## 4. Discussion

Alcoholics represent a risk group for viral hepatitis. Chronic alcohol consumption markedly worsens the prognosis of hepatitis. In patients with chronic HCV infection, even each standard drink (~12 g ethanol/day) increases the risk of cirrhosis, decompensation, and liver-related death by approximately 11% [22]. Similar effects are observed in chronic HBV infection. Heavy alcohol use substantially raises the risk of cirrhosis and hepatocellular carcinoma compared to HBV alone [23].

Data on the viral hepatitis prevalence in patients with alcohol dependence are inconsistent. While some seroepidemiological studies indicated that alcoholic patients showed a higher prevalence of viral hepatitis markers compared to the general population [24,25,26], others have reported prevalence rates that do not differ from those observed in the general population [27]. The differences are mainly observed for HBV and HCV, with higher prevalence in alcoholics due to shared risk factors (e.g., IDU) [28].

In 2021, Croatian households allocated a comparatively large proportion of their total consumption expenditure to alcoholic beverages (approximately 3.5%), placing Croatia among the highest-spending EU countries in this category, well above the EU average of 1.8% reported by Eurostat. Furthermore, alcohol consumption levels in Croatia are relatively high. Annual per capita pure alcohol intake among individuals aged 15 and over is estimated at approximately 9.2 L, and the country ranks among EU Member States with a notably high prevalence of daily alcohol consumption among adults [15].

Many seroepidemiological studies conducted in Croatia analyzed the prevalence of viral hepatitis in different exposed and non-exposed populations. However, data on the seroprevalence of viral hepatitis in alcoholics are scarce and mainly limited to HBV and HCV [8,29,30]. The prevalence of HAV in these populations was not investigated. Additionally, recent studies on the prevalence and potential risk factors for HBV, HCV, and HEV infection in alcohol abusers are lacking.

In the present study, the HAV seroprevalence in alcoholics (15.2%) was lower than that reported in the Croatian general population tested between 2008 and 2011 (41.6 and 40.5%) [17,31]. However, seroprevalence was similar to that observed in the comparable age control group tested in this study in the same period (14.5%). As in Croatia, many European countries have observed a long-term decline in HAV seroprevalence, largely attributed to improved hygiene and socioeconomic development, particularly among younger populations [32,33,34]. HAV seroprevalence largely reflects endemicity and age. In high-endemic areas, >90% of adults in both alcoholic and general populations are anti-HAV positive [28]. In regions with moderate or low HAV endemicity, seroprevalence is lower among younger adults; however, after adjustment for age and exposure history, no substantial differences are generally observed between alcoholic patients and the general population [35]. In our study, retired patients demonstrated a significantly higher HAV seroprevalence (46.2%) than employed (11.1%) and non-employed patients (17.6%). Retired and older individuals have had longer lifetime potential exposure. In addition, many chronic alcoholics are more likely to experience poor sanitation or hygiene, homelessness, or institutional living, which increases HAV transmission, especially earlier in life. In many countries transitioning from high to intermediate/low HAV endemicity, anti-HAV seropositivity rises steadily with age and peaks in adults aged ≥60 years, consistent with higher exposure risk in the past [33].

Some of the HBV studies in alcoholics date back to the 1980s–1990s and were conducted in different countries, where sociodemographic characteristics, local HBV prevalence, vaccination coverage, and risk behaviors (such as IDU or unsafe sexual practices) differ substantially, making direct comparisons challenging. Hospitalized alcoholics typically show higher rates of HBV markers than alcoholic outpatients [36], which may reflect selection bias. Patients with liver disease are more likely to be tested, or it may indicate a genuinely higher risk within this subgroup. In a Croatian cohort of alcoholic patients, the prevalence of naturally acquired HBV immunity (anti-HBs/anti-HBc positive) was similar to the control group (2.6 and 2.7%, respectively); however, it was lower compared to a 2011–2012 study (7.0%) [17]. None of the patients and controls were HBsAg positive. In a study conducted among prisoners (2005–2007), the prevalence of HBsAg and anti-HBc in alcoholics was 2.1% and 6.7%, respectively [29].

A systematic review combining data from multiple studies reported a weighted average HCV prevalence of approximately 16.3% in individuals with alcohol use disorders. Estimates varied substantially across studies, ranging from about 2.1% to 51%. Among alcohol-dependent individuals without a history of IDU or without advanced liver disease, HCV prevalence was lower, like that of the general population [27]. Many of the early high-prevalence reports likely included people with additional risk factors, such as IDU, past transfusion, and unstable housing, which confounds the association between alcohol use per se and viral hepatitis. Therefore, the results should be interpreted cautiously, as they do not uniformly reflect all individuals with alcohol use disorders. More recent, better-controlled studies that exclude IDU reported lower HCV prevalence, often close to that of the general population, indicating that alcohol use without additional risk behaviors may not significantly increase susceptibility to HCV. The HCV seroprevalence observed in this study (4.0%) is slightly above that reported in a previous regional Croatian study conducted in Istria County in 2013 (2.4%) among the same population group [30], and higher than the seroprevalence reported in the general population (0.9%) [8]. Comparing the anti-HCV and HCV RNA positivity among alcoholics and the control group in the present study, the difference between alcoholic patients and controls was statistically significant. No HCV-positive individuals were detected in the control group.

HEV seroprevalence is strongly influenced by age, geographic region, and zoonotic or dietary exposure; when these factors are considered, rates in alcoholics are often comparable to those observed in the general population [37]. The HEV seroprevalence detected in Croatian alcoholic patients was higher (14.6%) than that of a study from 2014 to 2015 (8.9%) [38]. However, no difference was found between patients and controls (13.6%). Anti-HEV seroprevalence in alcoholics was associated with the educational level, declining from 40.0% in patients with primary education to 5.3% in patients with university degrees. Lower HEV seroprevalence among alcoholic patients with university education compared with those with elementary or high school education can be explained by a combination of socioeconomic, behavioral, and environmental factors. Educational level is a strong proxy for socioeconomic status. Individuals with university education are more likely to have better housing and sanitation, whereas those with lower educational attainment are more likely to have been exposed to unsafe water sources, overcrowding, or poor sanitary conditions, thereby increasing lifetime exposure and seroprevalence. In addition, people with higher education may have greater awareness of food safety, consume food from regulated sources, and avoid high-risk dietary practices more often than those with lower education. Furthermore, individuals with a university education are generally more likely to seek medical care, screening, and counseling. Lower educational attainment is often associated with manual or agricultural work and more frequent contact with animals, livestock, or contaminated environments.

There are some limitations of this study that need to be addressed. There was a small number of patients and controls (only one clinical hospital center was included), which resulted in wide confidence intervals for some of the tested parameters. A relatively small cohort from a single center may be subject to selection bias. Therefore, the results should be interpreted with caution. In addition, it may have limited the ability to detect significant associations and may not fully represent the broader population. Due to the small number of HBV- and HCV-positive patients, predictors were analyzed only for HAV and HEV. The use of self-reported questionnaire data may be affected by reporting bias. Furthermore, data on alcohol-related risk behaviors were available for patients, but not for the control group.

## 5. Conclusions

In this Croatian cohort, patients treated for alcohol abuse showed a significantly higher seroprevalence of HCV compared with the general population, while the prevalence of HAV, HBV, and HEV markers did not differ between groups. HAV seropositivity was strongly associated with sociodemographic factors, particularly retirement status and the age at which occasional alcohol consumption started, suggesting cumulative exposure over time. HEV seroprevalence appeared to be influenced by educational level, with higher education conferring a protective effect. These findings highlight the continued relevance of targeted HCV screening among alcohol-dependent patients and support the need for tailored preventive strategies, including HAV vaccination and improved awareness of HEV risk factors in this vulnerable population. However, due to the small number of participants from a single center, future studies involving larger multicenter cohorts of alcoholic patients are needed to confirm our observations and the relationship between viral hepatitis and alcohol use.

## Figures and Tables

**Table 1:** Sociodemographic characteristics of patients included in the study.

Characteristic		N (%) Tested
Sex	Male	128 (84.8)
	Female	23 (15.2)
Age group	<40 years	32 (21.2)
	40–59 years	96 (63.6)
	60+ years	23 (15.2)
Educational level (Missing: 1; 0.6%)	Primary education	5 (3.3)
	High school	107 (70.9)
	University	38 (25.2)
Employment status (Missing: 22; 14.6%)	Employed	99 (65.5)
	Non-employed	17 (11.3)
	Retired	13 (8.6)

**Table 2:** Alcohol-related risk predictors and eating habits of patients included in the study.

Characteristic		N (%) Tested
First alcohol consumption (age) (Missing: 4; 2.7%)	<14 years	29 (19.2)
	14–18 years	80 (53.0)
	>18–22 years	28 (18.5)
	>22 years	10 (6.6)
Occasional alcohol consumption (age) (Missing: 3; 2.0%)	14–18 years	39 (25.8)
	>18–22 years	52 (34.4)
	>22–25 years	57 (37.8)
Period of frequent alcohol consumption (Missing: 2; 1.3%)	<5 years	36 (23.8)
	5–10 years	37 (24.5)
	>10–15 years	35 (23.2)
	>15 years	41 (27.2)
Alcohol type (Missing: 1; 0.6%)	Beer	49 (32.5)
	Wine	15 (9.9)
	Whiskey	37 (24.5)
	Combined	49 (32.5)
Quantity of alcohol consumed/24 h	≤1 L	16 (10.6)
	2–3 L	38 (25.2)
	>3 L	97 (64.2)
Number of treatments for alcoholism (Missing: 5; 3.3%)	First	99 (65.6)
	Second	23 (15.2)
	Third or more	24 (15.9)
Traveling	History of traveling/ long staying abroad	26 (17.2)
Eating habits	Consumption of game meat	91 (60.3)
	Consumption of pork liver	107 (70.9)

**Table 3:** ELFA tests for screening viral hepatitis markers.

Hepatitis Marker	Kit Name	Reference Range
Anti-HAV	VIDAS Anti-HAV Total	mIU/mL < 15 negative; 15–20 borderline; >20 positive
HBsAg	VIDAS HBs Ag Ultra	Test value <0.13 negative; ≥0.13 positive
Anti-HBs	VIDAS Anti-HBs Total II	mIU/mL <8 negative; ≥8–<12 equivocal; ≥12 positive
Anti-HBc	VIDAS Anti-HBc Total II	Index <1 negative; 1–1.4 equivocal; >1.4 positive
Anti-HCV	VIDAS Anti-HCV	Test value <1.00 negative; ≥1.00 positive
HEV IgG	VIDAS Anti-HEV IgG	U/mL <0.56 negative; ≥0.56 positive

HAV = Hepatitis A virus, HBsAg = Hepatitis B virus surface antigen, Anti-HBs = Antibodies to hepatitis B surface antigen, Anti-HBc = Hepatitis B core antibodies, Anti-HCV = Hepatitis C virus antibodies, HEV = Hepatitis E virus.

**Table 4:** Prevalence of viral hepatitis markers in patients and controls.

Virus	Viral Marker	Patients (*n* = 151)		Controls (*n* = 110)		*p*
		N (%) Positive	95% CI	N (%) Positive	95% CI	
Hepatitis A	Anti-HAV	23 (15.2)	9.9–21.9	16 (14.5)	8.5–22.5	0.877
Hepatitis B	HBsAg	0 (0)	NA	0 (0)	NA	NA
	Anti-HBs	18 (11.9)	7.2–18.2	22 (20.0)	12.9–28.7	0.073
	Anti-HBc/anti-HBs	4 (2.6)	0.7–6.6	3 (2.7)	0.6–7.7	0.969
Hepatitis C	Anti-HCV	6 (4.0)	1.5–8.5	0 (0)	NA	0.042
	HCV RNA	1 (0.6)	<0.1–3.5	0 (0)	NA	0.410
Hepatitis E	Anti-HEV	22 (14.6)	9.4–21.2	15 (13.6)	7.8–21.5	0.830

NA = Not applicable.

**Table 5:** Global association and likelihood ratio test results for anti-HAV and anti-HEV seropositivity.

Predictor	Global Association Test (*p*)		Global Likelihood Ratio Test (*p*)	
	Anti-HAV	Anti-HEV	Anti-HAV	Anti-HEV
Sex	1.000	0.755	0.678	0.754
Age (years)	<0.001	0.189	<0.001	0.175
Educational level	0.598	0.054	0.783	0.057
Employment status	0.007	0.410	0.014	0.537
Time of first alcohol consumption (age)	0.127	0.653	0.085	0.598
Time of occasional alcohol consumption (age)	0.006	0.975	0.002	0.975
Period of frequent alcohol consumption	0.117	0.082	0.095	0.103
Alcohol type	0.633	0.778	0.609	0.756
Quantity of alcohol consumed/24 h	0.542	0.212	0.530	0.243
Number of treatments	0.108	0.933	0.154	0.903
History of traveling Consumption of game meat/pork liver	0.767	0.768	0.553	0.621
	0.389	0.903	0.393	0.903

**Table 6:** Univariable logistic regression for anti-HAV and anti-HEV seropositivity (variables with statistical significance).

Viral Marker	Characteristic		OR	95% CI	*p*
Anti-HAV	Age	<40 years	Ref.		
		40–60 years	11.154	1.416–1440.474	0.015
		60+ years	49.400	5.548–6547.272	<0.001
	Employment status	Employed	Ref.		
		Non-employed	1.714	0.425–6.921	0.449
		Retired	6.857	1.949–24.119	0.002
	Age at the time of occasional alcohol consumption	14–18 years	Ref.		
		>18–22 years	5.911	0.696–50.206	0.103
		>22–25 years	13.571	1.710–107.685	0.013
Anti-HEV	Educational level	Primary school	Ref.		
		High school	0.303	0.047–1.948	0.208
		University	0.083	0.008–0.820	0.033

## Data Availability

The original contributions presented in the study are included in the article; further inquiries can be directed to the corresponding authors.

## Abbreviations

The following abbreviations are used in this manuscript:

HAV	Hepatitis A virus
HBV	Hepatitis B virus
HCV	Hepatitis C virus
HEV	Hepatitis E virus
WHO	World Health Organization
EU/EEA	European Union/European Economic Area
CI	Confidence interval
IQR	Interquartile range
OR	Odds ratio
ELFA	Enzyme-linked fluorescent assay
PCR	Polymerase chain reaction
ORF	Open reading frame
IDU	Intravenous drug use
